# When Testosterone Fades: Leydig Cell Aging Shaped by Environmental Toxicants, Metabolic Dysfunction, and Testicular Niche Crosstalk

**DOI:** 10.3390/cells15020158

**Published:** 2026-01-15

**Authors:** Aris Kaltsas, Fotios Dimitriadis, Athanasios Zachariou, Sotirios Koukos, Michael Chrisofos, Nikolaos Sofikitis

**Affiliations:** 1 Third Department of Urology, Attikon University Hospital, School of Medicine, National and Kapodistrian University of Athens, 12462 Athens, Greece; ares-kaltsas@hotmail.com (A.K.); mchrysof@med.uoa.gr (M.C.); 2 Department of Urology, Faculty of Medicine, School of Health Sciences, Aristotle University of Thessaloniki, 54124 Thessaloniki, Greece; helabio@yahoo.gr; 3 Laboratory of Spermatology, Department of Urology, Faculty of Medicine, School of Health Sciences, University of Ioannina, 45110 Ioannina, Greece; azachariou@uoi.gr (A.Z.); sotiriskoukos@gmail.com (S.K.)

**Keywords:** Leydig cells, testosterone, testicular aging, late-onset hypogonadism, endocrine-disrupting chemicals, autophagy, mitophagy, cellular senescence, obesity-related hypogonadism, single-cell RNA sequencing

## Abstract

Declining Leydig cell steroidogenesis contributes to late-onset hypogonadism and to age-associated impairment of male reproductive health. Determinants of dysfunction extend beyond chronological aging. This review synthesizes recent experimental and translational evidence on cellular and molecular processes that compromise Leydig cell endocrine output and the interstitial niche that supports spermatogenesis. Evidence spanning environmental endocrine-disrupting chemicals (EDCs), obesity and metabolic dysfunction, and testicular aging is integrated with emphasis on oxidative stress, endoplasmic reticulum stress, mitochondrial dysregulation, apoptosis, disrupted autophagy and mitophagy, and senescence-associated remodeling. Across model systems, toxicant exposure and metabolic stress converge on impaired organelle quality control and altered redox signaling, with downstream loss of steroidogenic capacity and, in some settings, premature senescence within the Leydig compartment. Aging further reshapes the testicular microenvironment through inflammatory shifts and biomechanical remodeling and may erode stem and progenitor Leydig cell homeostasis, thereby constraining regenerative potential. Single-cell transcriptomic atlases advance the field by resolving Leydig cell heterogeneity, nominating subsets that appear more vulnerable to stress and aging, and mapping age-dependent rewiring of interstitial cell-to-cell communication with Sertoli cells, peritubular myoid cells, vascular cells, and immune cells. Many mechanistic insights derive from rodent in vivo studies and in vitro platforms that include immortalized Leydig cell lines, and validation in human tissue and human clinical cohorts remains uneven. Together, these findings frame mechanistically informed opportunities to preserve endogenous androgen production and fertility through exposure mitigation, metabolic optimization, fertility-preserving endocrine stimulation, and strategies that target inflammation, senescence, and regenerative capacity.

## 1. Introduction

Leydig cells are the principal steroidogenic cells of the testicular interstitium and a central determinant of male reproductive endocrinology across mammalian species, including humans, and are commonly used rodent models. By producing testosterone, Leydig cells sustain intratesticular androgen concentrations required for normal spermatogenesis and also contribute to systemic androgen exposure that supports sexual function, body composition, and metabolic homeostasis [1,2]. Beyond testosterone, mature Leydig cells constitutively secrete insulin-like factor 3 (INSL3), a peptide hormone increasingly recognized in human endocrine studies as a complementary biomarker of Leydig cell differentiation status and functional reserve, distinct from the acute hypothalamic–pituitary regulation that shapes circulating testosterone dynamics [3,4]. Leydig cell biology is therefore not limited to “testosterone output” but encompasses developmental origin, niche dependence, endocrine and paracrine signaling, and stress adaptability across the male lifespan.

Aging of the male reproductive axis is typically gradual rather than abrupt, yet population studies consistently indicate that circulating testosterone concentrations decline with advancing age in many men, with a subset developing symptomatic late-onset hypogonadism (LOH) [5,6,7]. These endocrine changes occur in parallel with age-associated deterioration of reproductive health, including reduced fertility potential and increased vulnerability to reproductive pathologies [8,9]. Importantly, testosterone deficiency in clinical practice is heterogeneous: symptoms are nonspecific, biochemical thresholds vary across guidelines, and comorbidities such as obesity and chronic disease substantially modify androgen trajectories and clinical phenotypes [6,7,10]. Consequently, Leydig cell aging should be framed as a multidimensional process influenced by intrinsic cellular mechanisms and by systemic and environmental modifiers.

The relationship between advancing age and Leydig cell function is also mechanistically complex. Experimental work predominantly drawn from rodent aging paradigms and Leydig cell culture systems, together with integrative reviews, supports the concept that declining testosterone with age is driven predominantly by reduced testicular steroidogenic capacity rather than by a uniform fall in gonadotropin drive, as luteinizing hormone (LH) levels may remain stable or rise modestly in aging males [2,11]. Human tissue-based analyses further underscore that “Leydig cell aging” cannot be equated simply with irreversible steroidogenic failure of individual cells. For example, comprehensive histological and functional assessment of testes from men across age groups has reported a reduction in Leydig cell number and INSL3 expression with age, while the androgenic potential of cultured testicular tissue fragments did not show an obligatory age-dependent collapse, highlighting the potential contribution of cellular composition, differentiation state, and microenvironmental context [3,12]. These observations reinforce the need to dissect Leydig cell aging at cellular, molecular, and niche levels rather than relying solely on serum testosterone as a surrogate, and to interpret mechanistic findings with attention to species and strain differences in testicular physiology when considering translational relevance.

In contemporary settings, Leydig cell aging increasingly reflects “combined-hit” biology, in which chronological aging interacts with exposures and metabolic states that accelerate stress responses. Endocrine-disrupting chemicals (EDCs) are widely prevalent and have been associated with adverse male reproductive outcomes in experimental and human studies, with effects that plausibly involve Leydig cell steroidogenesis and stress signaling [13]. In parallel, obesity-related secondary hypogonadism has emerged as a common, potentially reversible clinical entity, characterized by disrupted hypothalamic–pituitary–gonadal signaling and impaired testicular endocrine function, often improving with substantial weight loss [14]. These modifiers are clinically relevant because they can compress the timeline of functional decline, amplify inflammometabolic stress, and blur boundaries between “physiological” aging and acquired Leydig cell dysfunction [15,16]. Interpretation is complicated by heterogeneity in exposure paradigms, since mechanistic studies may use doses, mixtures, routes, or acute windows that do not mirror environmentally relevant human exposures, and outcomes can shift with timing, particularly between developmental windows and adult exposure. Species and strain differences add further variability that can shape baseline steroidogenic capacity and stress susceptibility.

**Figure 1:**
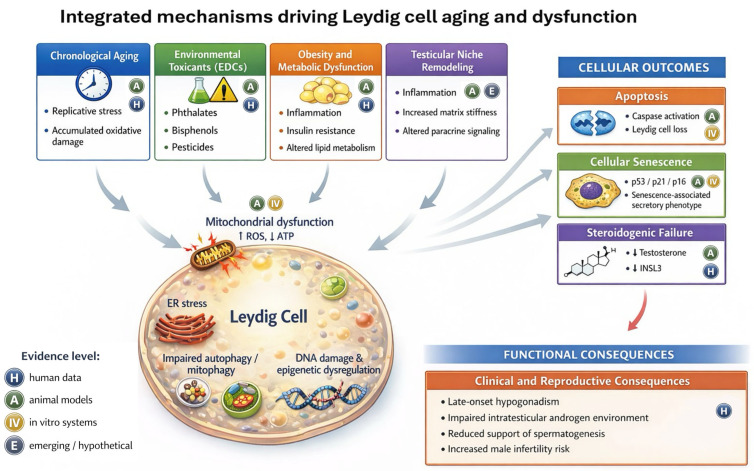
Integrated framework for Leydig cell aging. Drivers and accelerators including chronological aging, endocrine-disrupting chemicals (EDCs), metabolic dysfunction, and interstitial niche remodeling converge on shared mechanistic hubs including mitochondrial and redox stress, endoplasmic reticulum (ER) stress, impaired autophagy and mitophagy, and DNA damage responses. Arrows indicate mechanistic convergence and regulatory crosstalk rather than a strict temporal sequence. Evidence strength differs across pathways, with several nodes supported predominantly by rodent in vivo and Leydig cell-based in vitro studies and more limited confirmation in human testicular tissue and clinical cohorts. Downstream Leydig cell state changes include reduced steroidogenic machinery and context-dependent apoptosis and or senescence, with consequent niche-level effects and mechanism-informed intervention opportunities.

Despite the breadth of prior work on Leydig cell steroidogenesis, aging, and EDCs, key gaps remain. The evidence base is fragmented across stressors, endpoints, and experimental platforms, with inconsistent model systems and exposure paradigms that limit cross study comparison and constrain translational inference. Human confirmation is limited for several proposed mechanistic hubs, and clinically usable biomarkers that reflect Leydig cell differentiation and stress state beyond serum testosterone remain limited. This narrative review adds value beyond prior syntheses by integrating intrinsic aging with environmental and metabolic stressors through a convergence model of shared cellular stress pathways and testicular niche crosstalk. It incorporates explicit model context labeling and exposure timing considerations to distinguish mechanisms supported by human data from those inferred primarily from animal and cell culture studies. It also uses mechanism-linked translational framing to align causal claims with evidence strength and to define realistic opportunities for pathway-targeted intervention. The objectives are to synthesize Leydig cell heterogeneity and interstitial niche dependence across the male lifespan, to connect systemic modifiers with local steroidogenic capacity, and to clarify opportunities and boundaries for clinical translation. Figure 1 summarizes the integrated framework proposed.

## 2. Review Type and Literature Search Strategy

This article is a narrative review that synthesizes experimental, translational, and clinical evidence on Leydig cell aging and dysfunction across the male lifespan. The focus is on convergence between chronological aging, endocrine disrupting chemical exposures, metabolic dysfunction, and testicular niche crosstalk through shared cellular stress pathways. A narrative review design was selected because the evidence spans diverse experimental systems, exposure paradigms, and clinical endpoints. This heterogeneity limits meaningful effect size synthesis and favors conceptual integration that supports translational interpretation.

An iterative, topic-driven search of the English-language literature was conducted in PubMed and MEDLINE. Embase, Scopus, and Web of Science Core Collection were also searched. Targeted searches in Google Scholar were used to capture emerging work. Reference lists from key papers were screened manually to identify seminal studies and recent advances. Evidence from 2015 to 2025 was prioritized to balance mechanistic foundations with field recency. Particular emphasis was placed on work from 2021 to 2025 that has reshaped current concepts. This includes single-cell testis atlases, refined senescence frameworks, and newer exposure paradigms. The earlier literature was included selectively when it defined core principles of Leydig cell development and regulation or established canonical experimental models. Foundational mechanistic observations were retained when they remain central to interpretation of contemporary data.

Search strings used Boolean operators and combined terms related to Leydig cell biology and steroidogenesis. Testosterone was included as a core term. Additional terms captured aging and senescence, oxidative stress and mitochondria, and autophagy and mitophagy. Terms related to EDCs were included along with major toxicant classes. The search also included obesity, metabolic syndrome, diabetes, inflammation, testicular niche, and single-cell transcriptomics. These keyword clusters were refined iteratively to align with shared mechanistic hubs and to capture overlap between stress responses, niche signaling, and steroidogenic dysfunction.

Eligible sources included peer-reviewed primary studies in vivo and in vitro. Human observational and clinical studies were included when available. Translational research was included when it directly addressed Leydig cell steroidogenesis, stress responses, or microenvironmental regulation. Non-English sources and conference abstracts without full-text publication were excluded. Studies were deprioritized when Leydig cell-specific outcomes were absent or when exposure paradigms and endpoints were described insufficiently for mechanistic interpretation. Exposure context was considered during interpretation with attention to dose, duration, and timing. Environmentally relevant exposures were distinguished from high-dose experimental paradigms when discussing mechanistic endpoints. Interpretation also noted species, strain, and cell system differences, and it appraised dose realism and exposure timing to support cautious translation.

Review articles were used to map the field, reconcile terminology, and identify key primary studies. When review articles informed a statement, the underlying primary reports were traced and appraised directly whenever possible. Mechanistic statements were preferentially anchored to primary data when possible. Human data were prioritized to frame translational relevance. Animal- and cell-based studies were used to delineate mechanisms and to identify areas where evidence remains inferential. No formal risk of bias scoring was applied because of the narrative design. This strategy may amplify selection and publication biases, and it limits formal quantification of uncertainty across heterogeneous outcomes. The English-language restriction and the iterative nature of the search may also limit completeness. Heterogeneity in species and strain is addressed within the relevant sections. The same approach is used for cell systems, exposure paradigms, and outcome measures to contextualize consistency, limitations, and translational readiness.

## 3. Environmental Toxicants and Leydig Cell Injury

A substantial body of research now implicates environmental toxicants, particularly EDCs, as modifiers of Leydig cell steroidogenesis and male reproductive health [17]. EDCs are exogenous compounds that interfere with hormonal signaling and are ubiquitous in modern environments [18,19]. Common classes include phthalates, bisphenols such as bisphenol-A (BPA), selected pesticides, and industrial byproducts. Mechanistic evidence is derived mainly from rodent in vivo studies and Leydig cell-based in vitro systems, where exposures are frequently associated with reduced steroidogenic output and activation of cellular stress programs, whereas direct validation in human testicular tissue remains uneven [13]. Accordingly, proposed contributions of EDCs to population-level reproductive trends should be interpreted as plausible and multifactorial rather than singularly causal, with inference shaped by mixture effects, exposure timing, and species and strain differences [20].

### 3.1. Study Models and Exposure Paradigms for Interpreting Toxicant Effects

Interpretation of toxicological evidence demands careful consideration of the study models and exposure paradigms used. Mechanistic insights into Leydig cell toxicant effects largely derive from rodent in vivo studies and in vitro experiments using primary or immortalized Leydig cells. Each model has inherent limitations. Immortalized Leydig cell lines (such as the mouse TM3 or MA-10) enable long-term experiments but often differ from primary cells in key functions—for example, some tumor-derived lines do not produce testosterone as an end product of steroidogenesis and may proliferate or undergo stress responses uncharacteristic of normal cells. Primary adult Leydig cells, on the other hand, closely reflect in vivo physiology and respond to LH, but they survive and secrete testosterone for only a short period ex vivo (on the order of days), which limits their utility for chronic exposure studies. Species differences are also important: the regulation of testicular steroidogenesis and the susceptibility to toxicants can vary between rodents and humans [21]. Even among rodent strains, baseline testosterone levels and antioxidant defenses differ, meaning one strain might experience Leydig cell oxidative injury or apoptosis where another shows resilience.

Another crucial factor is dosing and exposure regimen. Animal studies often utilize exposure levels that exceed typical human exposures by orders of magnitude. For instance, anti-androgenic effects of phthalates in rodents are frequently reported at doses of 10–100 mg/kg/day, far above the low environmental doses humans encounter [22]. Conversely, real-world human exposure is typically chronic and involves complex mixtures of chemicals rather than single agents. Such mixtures can have additive or even synergistic effects that do not follow a classic linear dose–response; notably, low-dose combinations of EDCs have disrupted Leydig cell function in experimental models even when each component alone was at a subthreshold level [23]. Timing of exposure further modulates outcomes. Developmental exposures—for example, in utero or during puberty—can have lasting impacts on the formation and function of the Leydig cell population that differ from the effects of adult-onset exposure. Some toxicants predominantly affect fetal or neonatal Leydig cells, leading to a reduced pool of these cells in adulthood without necessarily causing acute cell death at the time of exposure. In adult models, prolonged low-level exposures might induce subtler changes such as diminished steroidogenic capacity or early onset of cellular senescence rather than overt cell loss. Finally, endpoint heterogeneity complicates comparisons across studies: different experiments measure different indicators of Leydig cell injury (e.g., apoptosis rates, hormone levels, or expression of stress response genes), and not all endpoints are equally sensitive [20]. One study might report significant apoptosis, whereas another using a lower dose or focusing on a functional endpoint finds only moderate hormonal changes with no obvious cell death. These methodological differences can lead to negative or mixed results in the literature. In summary, careful appraisal of the experimental context—including model system, dose and duration, mixture complexity, life stage of exposure, and endpoints assessed—is necessary when extrapolating toxicant findings toward human risk. This framework underscores why integrating evidence from multiple models and pursuing more human-relevant exposure studies are priorities for the field [24].

### 3.2. Apoptosis as a Terminal Outcome in Leydig Cell Toxicology

Apoptosis represents a terminal programmed cell death outcome in Leydig cells after toxicant injury, and it reflects definitive loss of cellular viability rather than an adaptive stress response. In rodent exposures and Leydig cell-based assays, EDCs have been shown to activate apoptotic signaling beyond physiological turnover, supporting apoptosis as a late-stage endpoint of endocrine disrupting injury [20,25]. This death program can proceed through death receptor signaling or through an intrinsic pathway driven by mitochondrial dysfunction, and in these models, current evidence most often implicates the intrinsic mitochondrial route in Leydig cell injury after endocrine-disrupting exposures [26]. This pattern aligns with the central role of mitochondrial integrity in steroidogenesis and redox balance, placing oxidative stress and mitochondrial dysfunction upstream of the terminal apoptotic outcome. At the tissue level, sustained apoptosis would be expected to reduce functional Leydig cell mass and compromise androgen output, providing a translational link between subcellular injury and endocrine phenotypes.

In Leydig cell-based in vitro models, benzo[b]fluoranthene (BbF), a polycyclic aromatic hydrocarbon, has been shown to activate the p53 tumor suppressor pathway. The effect has been linked to inhibition of the Akt-mediated Mdm2 signaling axis that normally restrains p53, followed by increased Bax expression, activation of caspase 3, and apoptotic cell death [27]. Similarly, BPA also induces apoptosis in Leydig cell models and has been linked to epigenetic and transcriptional changes. BPA exposure markedly suppresses the expression of TET1, an enzyme involved in DNA hydroxymethylation. It also suppresses several calcium channel genes. TET1 supports Leydig cell proliferation and survival, and its downregulation may contribute to BPA-associated proapoptotic effects [28]. These examples highlight that EDCs can converge on mitochondrial apoptotic signaling through distinct upstream inputs, including p53-linked stress signaling and epigenetic disruption that may lower the threshold for mitochondrial injury. Several mechanistic details remain unresolved, including the relevant cell surface receptors and the gene networks that drive vulnerability or resilience. Interpretation is constrained by heterogeneity in exposure timing and dose selection across rodent studies and in vitro assays. Species and strain differences may also shape basal steroidogenic set points and stress responses, contributing to variable apoptosis susceptibility across models. Endpoints vary across studies, ranging from transcript shifts to caspase activation, which complicates comparisons between studies and limits inference about sustained Leydig cell depletion in vivo.

### 3.3. Autophagy and Mitophagy as Stress Adaptive Quality Control That Modulate Apoptosis

Autophagy and mitophagy are regulated quality control processes that usually support survival during stress rather than acting as primary death programs. Basal autophagy in Leydig cell models contributes to homeostasis through removal of damaged organelles and proteins [29]. Under toxicant exposure, autophagy often buffers mitochondrial injury and can restrain intrinsic apoptotic signaling, which is consistent with a stress adaptive role [30]. Autophagy contributes to cell death mainly when oxidative stress and mitochondrial dysfunction exceed coping capacity or when autophagic flux is disrupted. Toxicants may induce autophagy as a compensatory response. They may also impair autophagic flux. Either pattern can influence whether cells recover or progress to apoptosis.

BPA has been reported in Leydig cells to induce autophagy through suppression of the protein kinase B (AKT)/mechanistic target of rapamycin (mTOR) signaling pathway, a negative regulator of autophagy [31]. This mechanism links BPA to the autophagy and mitophagy imbalance hub and can shape mitochondrial quality control that modulates apoptosis. Lead exposure also increases expression of autophagy-related proteins such as Beclin-1 and LC3 in Leydig cell systems, which is interpreted as a response that attempts to limit injury [32]. Such marker induction is compatible with stress responses driven by oxidative stress and mitochondrial dysfunction. Other toxicants appear to increase vulnerability by disrupting autophagic flux. Tributyltin chloride induces endoplasmic reticulum stress in Leydig cell models and suppresses autophagic flux, a combination associated with increased apoptosis and cell cycle arrest [33]. Cadmium exposure triggers Leydig cell apoptosis at least in part by inhibiting mitophagy, which permits accumulation of dysfunctional mitochondria and promotes intrinsic apoptotic signaling [34]. These findings underscore crosstalk between apoptosis and autophagy in determining Leydig cell fate under toxicant exposure. In several experimental models, intact autophagy attenuates toxicant-induced apoptosis, while disruption of autophagic flux shifts the balance toward cell death [20].

Targeted modulation of autophagy has therefore been explored as a protective strategy in preclinical settings. In preclinical Leydig cell models, Vitamin D_3_ supplementation has been shown to attenuate BPA toxicity by enhancing autophagy through activation of the vitamin D receptor pathway [35]. The reported protection is consistent with strengthening adaptive autophagy and mitophagy to counter mitochondrial dysfunction and reduce downstream apoptotic signaling. Prenatal exposure to phthalates has also been reported to trigger an autophagic protective response in adult rat Leydig cells through epigenetic regulatory mechanisms [36]. The developmental window highlights that exposure timing can reprogram stress-adaptive pathways and may contribute to heterogeneous adult endpoints. Interpretation across toxicants is limited by variation in dose selection and exposure duration. Many studies rely on static marker endpoints that may not report autophagic flux. Species and strain differences in Leydig cell maturation and stress signaling further complicate translation to human tissue. Understanding these pathways could yield novel interventions to prevent Leydig cell loss.

### 3.4. Premature Leydig Cell Senescence and Redox-Driven Endocrine Dysfunction

Another consequence of chronic exposure to EDCs is the premature onset of cellular senescence in Leydig cells. Cellular senescence is characterized by permanent growth arrest and by profound metabolic and signaling alterations, including increased secretion of pro-inflammatory mediators. Within the testicular interstitium, this secretory shift can amplify inflammation and weaken paracrine support for steroidogenesis. Although senescence in Leydig cells normally develops as part of the aging process, environmental stressors can markedly accelerate its appearance [37,38]. Oxidative stress is a central driver of cellular aging, and many endocrine-disrupting chemicals, as well as related toxic exposures, induce excessive production of reactive oxygen species (ROS) within the testes [39]. Oxidative stress is therefore the upstream mechanistic hub that links these exposures to mitochondrial dysfunction, autophagy and mitophagy imbalance, inflammatory signaling, apoptosis, and senescence. This convergence provides a mechanistic bridge to endocrine dysfunction. Oxidative damage to biomolecules and telomere shortening can trigger DNA damage responses, activating p53/p21 or p16 pathways that halt the cell cycle and induce a senescent state [40,41]. Senescence markers are not uniform across systems, and their interpretation depends on the sampling window. In vitro Leydig cell models often emphasize early increases in p21 or p16 expression and senescence-associated beta galactosidase activity. Later in vivo sampling more consistently captures persistent secretory remodeling and sustained suppression of steroidogenic endpoints.

In in vivo animal models, chronic low-dose exposure to specific pollutants such as the diesel exhaust-derived compound 1-nitropyrene increases testicular indices of oxidative stress, including elevated malondialdehyde levels and depletion of glutathione [42,43]. These redox disturbances are accompanied by impaired steroidogenic function of Leydig cells, supporting oxidative stress as a proximal determinant of androgen output [44]. It is postulated that sustained oxidative stress induced by EDCs not only eliminates a subset of Leydig cells through apoptosis but also drives surviving cells into a senescent, androgen-deficient phenotype. Dose realism and exposure timing are important modifiers of this balance. Acute high-intensity insults can favor cell death, whereas chronic sublethal stress may preferentially stabilize senescence programs. Species and strain differences in antioxidant capacity may shift the oxidative stress threshold for apoptosis and for senescence. Endpoint heterogeneity also reflects differences in cell isolation, tissue compartment sampling, and the senescence markers used for readout. Recent experimental and translational work has refined this concept by demonstrating that EDC-induced Leydig cell dysfunction may manifest as apoptosis, senescence, or a combination of both, and that these processes can influence one another [45]. For example, the mycotoxin T-2 toxin was shown to cause oxidative stress in rat Leydig cells, leading to simultaneous markers of apoptosis and senescence [46].

Conversely, some toxicants predominantly induce autophagy and cellular senescence without triggering immediate apoptosis. Organophosphate flame retardants and related plasticizers can provoke oxidative stress, mitochondrial dysfunction, formation of autophagic vacuoles, and cell cycle arrest in testicular and Leydig cell models while leaving short-term cell viability largely preserved [47,48]. Here, oxidative stress again sits upstream of mitochondrial dysfunction and perturbed autophagy and mitophagy, supporting a route to senescence without immediate loss of viability. The dynamic interplay between senescence, autophagy, and cell death in Leydig cells exposed to environmental stressors has therefore emerged as a new frontier [49,50]. Ongoing research seeks to clarify how sublethal toxic insults chronically impair Leydig cell endocrine function through senescence-associated remodeling and altered secretory profiles, even when the cells remain viable [51]. From a translational perspective, preserved viability with senescence-associated secretory remodeling implies that Leydig cell number may not track endocrine capacity. This strengthens the case for endpoints that integrate redox state, mitochondrial function, senescence markers, and steroidogenic output within the same exposure paradigm.

### 3.5. Translational Implications and Leydig Cell-Directed Protective Strategies

From a translational perspective, exposure mitigation remains the most direct preventive strategy, whereas pharmacologic or nutraceutical “protection” is supported predominantly by preclinical evidence. Across rodent injury paradigms and Leydig cell-based systems, interventions that reduce oxidative injury or stabilize mitochondrial function are often accompanied by partial preservation of steroidogenic endpoints and attenuation of downstream apoptosis-associated readouts [52,53]. In an in vitro Leydig cell model exposed to hydrogen peroxide, rutin reduced ROS and lipid peroxidation and testosterone production, which is consistent with mitigation of redox-driven mitochondrial stress [54]. Icariin has been reported to reverse phthalate-induced suppression of Leydig cell proliferation and to decrease intracellular ROS, supporting a similar redox-centered mechanism that converges on oxidative stress and mitochondrial dysfunction in in vitro Leydig cell models [55,56]. In rat models of di-(2-ethylhexyl) phthalate (DEHP)-induced testicular injury, mangiferin preserved steroidogenic function in part by suppressing oxidative stress-driven apoptotic pathways in Leydig cells [57]. These findings are synthesized in recent reviews that highlight antioxidant strategies as candidate interventions to limit oxidative and apoptotic injury [37,58,59].

Another strategy under consideration involves direct modulation of the apoptotic machinery in Leydig cells. Upregulation of anti-apoptotic proteins such as BCL-2 and heat-shock proteins, together with pharmacological inhibition of key caspases, is being explored in preclinical models as a means to limit Leydig cell loss during toxicant exposure [60]. Consistent with this concept, one experimental study reported that protection against DEHP-induced testicular injury was accompanied by increased testicular HSP70 expression and reduced Leydig cell apoptosis, suggesting that induction of molecular chaperones may contribute to cytoprotection in this context [57]. Modulation of autophagy and mitophagy has also been proposed as a therapeutic avenue, since efficient mitochondrial quality control can attenuate intracellular stress and may prevent the intrinsic apoptotic cascade that many endocrine-disrupting chemicals appear to activate in Leydig cell models [34,61]. More broadly, delineating the precise molecular mechanisms by which endocrine-disrupting chemicals injure Leydig cells, including receptor interactions, downstream signaling cascades, mitochondrial pathways, and gene targets, is expected to reveal actionable drug targets for intervention [62]. Receptor interactions, downstream signaling cascades, mitochondrial pathways, and gene targets remain central to this effort. This is now an active area of translational research that seeks to develop pharmacological agents and evidence-based lifestyle recommendations to preserve Leydig cell function and support male fertility in the face of pervasive environmental toxicant exposure [20].

## 4. Aging, Obesity, and Metabolic Influences on Leydig Cell Function

### 4.1. Chronological Aging and Late-Onset Hypogonadism: Convergence of Cellular Stress Pathways

Beyond environmental chemicals, intrinsic factors such as chronological aging and metabolic health exert profound effects on Leydig cell function and testicular endocrine capacity [12,63]. Epidemiological and longitudinal studies indicate that a subset of men experience an age-associated decline in circulating testosterone, commonly framed clinically as LOH [64,65]. Declines in semen parameters are also observed with advancing paternal age, although these changes likely reflect combined germline and somatic contributions rather than Leydig cell biology alone [66]. Recent research indicates that Leydig cells are particularly vulnerable to aging processes in the testis [67]. Age-related Leydig cell dysfunction is multifactorial, involving both local cellular changes and systemic hormonal alterations. Primary causes identified in the pathogenesis of LOH include chronic oxidative stress, inflammation, mitochondrial dysfunction, and endoplasmic reticulum (ER) stress within the Leydig cells [8]. These stressors can impair the steroidogenic machinery of the cell—for example, oxidative damage may downregulate cholesterol transport and steroidogenic enzyme activity, while ER stress can disrupt protein folding of key enzymes. Over time, such insults accelerate Leydig cell senescence or death, leading to reduced testosterone biosynthesis [68].

### 4.2. Obesity, Metabolic Syndrome, and Acquired Leydig Cell Dysfunction

Metabolic disorders, especially obesity and metabolic syndrome, have emerged as significant accelerants of Leydig cell aging and dysfunction. Men with obesity often present with subnormal testosterone levels, and epidemiological studies show that obesity-related metabolic derangements correlate with increased risk of infertility [69]. The relationship between obesity and reduced testosterone levels is both bidirectional and complex. Obesity can diminish gonadotropin secretion through hypothalamic and pituitary mechanisms, thereby lowering LH output. At the same time, even when LH concentrations are normal or elevated, Leydig cells in obese individuals may exhibit a blunted functional response, indicating an acquired form of Leydig cell dysfunction [14]. A recent review underscored that obesity and related metabolic disturbances accelerate functional aging of Leydig cells, lower testosterone concentrations, and contribute to the development of LOH [68]. Adipose tissue-derived factors, including pro-inflammatory cytokines, elevated leptin, and increased estrogen generated through enhanced aromatase activity, are likely to contribute to the suppression of Leydig cell steroidogenesis. Chronic low-grade inflammation in obesity infiltrates even the testes, where activated immune cells and inflammatory cytokines can impair Leydig cell function and promote a senescent phenotype [68]. Altered lipid metabolism in obesity also appears to influence Leydig cell function. Because testosterone synthesis relies on adequate cholesterol availability and efficient lipid processing within Leydig cells, dyslipidemia or excessive intracellular lipid accumulation can interfere with normal steroidogenic activity. Consistent with this concept, obesity and insulin-resistant states are associated with heightened oxidative stress in Leydig cells and reduced expression of key genes involved in steroidogenic enzyme production [68]. Mitochondrial dysfunction has also been noted—the mitochondria in aging or obese testes show diminished capacity for energy production and steroid precursor metabolism, which hampers testosterone output [68]. Thus, metabolic health is intimately linked to Leydig cell vitality.

### 4.3. Ketogenesis and Ketone Signaling in Leydig Cell Aging

One particularly compelling new insight into the metabolic regulation of Leydig cell aging involves the role of ketogenesis and cellular energy supply within the testes. A landmark 2025 study by Liu and colleagues demonstrated that impaired production of ketone bodies within Leydig cells constitutes a critical mechanism driving testicular aging [67]. Using single-cell transcriptomic analysis, the authors demonstrated that Leydig cells in aged mice exhibit markedly reduced expression of Hmgcs2, the rate-limiting enzyme that mediates ketogenesis and enables the production of ketone bodies from fatty-acid substrates [67]. In keeping with this mechanism, the concentrations of the principal ketone bodies β-hydroxybutyrate and acetoacetate were markedly lower in aged testes compared to those of young animals [67]. This finding is significant because β-hydroxybutyrate functions not only as an energy substrate but also as a signaling metabolite. The study demonstrated that β-hydroxybutyrate enhances the expression of FOXO3a, a transcription factor that mitigates oxidative stress and supports cell survival, by promoting histone acetylation within Leydig cells [67]. This epigenetic mechanism aligns with prior evidence in rodents showing that β-hydroxybutyrate acts as a class I histone deacetylase inhibitor, which upregulates FOXO3a and enhances oxidative stress resistance [70].

In young Leydig cells, robust ketogenesis yields higher local β-hydroxybutyrate levels that help maintain FOXO3a activity. The resulting FOXO3a activation counteracts cellular senescence and supports normal testosterone synthesis. In aged or metabolically compromised Leydig cells, declining ketone production leads to inadequate FOXO3a activation. This loss of FOXO3a-mediated protection accelerates cellular senescence. Causality was confirmed by direct manipulation of ketogenesis in vivo. Silencing Hmgcs2 in young mouse Leydig cells triggered premature cellular aging. Conversely, enhancing ketone body availability either via genetic interventions or through oral β-hydroxybutyrate supplementation attenuated Leydig cell aging and improved testosterone levels in older mice [67]. This discovery directly links systemic metabolic fuel utilization to testicular aging. It indicates that metabolic syndrome, which is characterized by mitochondrial inefficiency and altered fatty acid metabolism, could similarly impair Leydig cell ketogenesis and accelerate functional decline. In this framework, obesity and metabolic syndrome can be viewed as creating an energy deficit within Leydig cells. The resulting lack of ketone-derived signals deprives the cells of protective metabolic input and increases their vulnerability to senescence. While these mechanistic insights are still emerging, they raise important translational questions, including whether dietary or pharmacological modulation of testicular energy metabolism could help preserve Leydig cell function in aging or obese men [67].

Importantly, the connection between ketogenesis and FOXO3a identified in these mouse studies [67] has been delineated only in rodents, and its status in human Leydig cell aging remains unconfirmed. FOXO transcription factors are well-established conserved mediators of cellular stress resistance across species. In humans, genetic variants in FOXO3a have been linked to exceptional longevity in centenarian cohorts, underscoring the evolutionary conservation of FOXO-driven stress responses [71]. Despite these hints of conservation, it is not yet known whether aging human Leydig cells indeed experience diminished ketone production or reduced FOXO3a activation as observed in mice. A recent single-cell transcriptomic atlas of human testes spanning ages 21 to 69 years provides a resource to examine whether HMGCS2 expression in Leydig cells declines with age or metabolic disease [72]. A complementary approach would be to perform targeted metabolomic studies measuring intratesticular ketone body levels in men across different ages or metabolic states. Such analyses would reveal whether a similar decline in β-hydroxybutyrate occurs with aging or metabolic syndrome, thereby testing if human Leydig cells undergo the same loss of protective ketone signaling. Validating this pathway in humans would guide potential interventions aimed at preserving Leydig cell function during aging.

### 4.4. Clinical Implications for Fertility, Spermatogenesis, and LOH Management

From a clinical standpoint, age-related Leydig cell decline affects not only androgen production but also male fertility. Older men frequently exhibit reduced sperm output and compromised sperm quality, and although this is partly attributable to intrinsic germ-cell aging, suboptimal intratesticular testosterone caused by declining Leydig cell function can further exacerbate the impairment [73]. Evidence from human testicular aging studies indicates that by the fifth decade of life, both Sertoli and Leydig cells display substantial transcriptomic alterations, with Leydig cells showing marked changes in pathways governing testosterone biosynthesis and metabolism [72]. Most available human datasets are cross sectional and clinically heterogeneous. Confounding by comorbidity burden, medication exposure, and variable tissue procurement can contribute to endpoint heterogeneity and limit causal inference. In practical terms, the aging testis undergoes a deterioration of the somatic environment required to support spermatogenesis, including hormonal and metabolic inputs [6,74]. These observations have stimulated increasing interest in managing LOH not only for symptomatic relief in older men but also within the broader context of fertility preservation for middle-aged men seeking paternity [5].

The current standard treatment for clinically significant hypogonadism is testosterone replacement therapy (TRT), yet exogenous testosterone is contraindicated in men who aim to maintain fertility because it suppresses gonadotropin secretion and can markedly reduce sperm production [10]. For men who are not pursuing fertility, often after completion of family building and commonly beyond reproductive age, TRT can be appropriate for symptom treatment after individualized risk benefit discussion and structured monitoring [75]. The degree and timing of spermatogenic suppression vary across testosterone formulations and doses, and recovery after cessation is heterogeneous [76]. This replacement approach bypasses Leydig cell steroidogenesis and does not directly address upstream mechanisms such as oxidative stress, mitochondrial dysfunction, inflammatory signaling, senescence, and disrupted autophagy and mitophagy. This creates a clear need for fertility-preserving alternatives, including selective estrogen receptor modulators and gonadotropin-based regimens such as human chorionic gonadotropin, that can maintain gonadotropin support of Leydig cell mitochondrial steroidogenesis and intratesticular testosterone in aging or metabolically compromised men without adversely affecting spermatogenesis [77].

### 4.5. Lifestyle Interventions and Adjunctive Metabolic Support

Lifestyle modification remains a first-line strategy for improving Leydig cell function. Weight reduction and improved metabolic control through dietary change and regular exercise have been shown to increase endogenous testosterone concentrations in overweight and obese men [78]. Even modest weight loss can reverse elements of endocrine dysregulation, and one study reported that each kilogram of weight lost was associated with a measurable rise in serum testosterone [79]. Weight reduction also diminishes systemic inflammation and lowers excessive estrogen production, thereby creating a hormonal milieu that is more favorable for optimal Leydig cell activity [80]. Some clinical studies have observed improvements in semen parameters following lifestyle-induced weight loss, although findings are variable and long-term effects on fertility require further investigation [81,82]. Moreover, a recent meta-analysis confirmed significant gains in sperm concentration and motility and a reduction in sperm DNA fragmentation following weight reduction [83]. However, results remain variable across studies, and the long-term effects on fertility outcomes are still being determined.

As an adjunct to lifestyle changes, metabolic therapies that promote weight loss and improve insulin sensitivity have shown promise for further enhancing Leydig cell function. For instance, a randomized trial compared weekly semaglutide, a weight loss medication, with testosterone replacement in obese men with functional hypogonadism. Semaglutide raised serum testosterone levels similar to testosterone injections but significantly improved normal sperm morphology and maintained sperm counts, whereas exogenous testosterone led to a decline in sperm concentration [84]. These findings suggest that targeting underlying metabolic dysfunction can restore endocrine balance and support spermatogenesis without the fertility-suppressing effects of direct testosterone administration.

In parallel, nutritional supplementation with antioxidants and anti-inflammatory compounds such as vitamin E, coenzyme Q10, and omega-3 fatty acids is being explored as a means to support testicular function in aging men and in individuals with metabolic syndrome [85]. Preclinical studies in obese or toxicant-exposed animal models and Leydig cell lines commonly report improvements in redox-related readouts alongside upregulation of selected steroidogenic transcripts and/or higher testosterone endpoints [86]. However, clinical evidence is heterogeneous and does not consistently demonstrate fertility or pregnancy benefit, underscoring that redox modulation observed in experimental systems may not translate into meaningful reproductive outcomes in unselected human cohorts [87]. This outcome underscores the need for cautious interpretation and suggests that antioxidant therapy may require a more targeted approach. Specifically, supplementation might benefit only those men with clear oxidative stress or micronutrient deficiencies, rather than being used routinely in all cases.

### 4.6. Emerging Anti-Aging Strategies: Senolytic Concepts

Another emerging concept is senolytic therapy, which involves selectively eliminating senescent cells. This approach remains experimental but has generated interest because clearing non-functional senescent cells could theoretically allow the regeneration of healthier Leydig cell populations. Compounds such as FOXO4-DRI peptides and inhibitors of the BCL-2 family have demonstrated senolytic activity in other tissues, prompting speculation that similar strategies might one day rejuvenate the Leydig cell compartment in cases of late-onset hypogonadism [68]. At present, however, no clinical trials have evaluated senolytics for male reproductive endocrinology, and concerns regarding safety must be addressed since senescent cells also serve physiological functions.

### 4.7. Fertility-Preserving Endocrine Pharmacotherapy: Gonadotropins and SERMs

A more immediately applicable therapeutic option in the context of fertility preservation is the use of gonadotropin stimulation or selective estrogen receptor modulators (SERMs) in place of exogenous testosterone [88,89]. Human chorionic gonadotropin can stimulate endogenous Leydig cell testosterone production and raise systemic hormone levels without compromising intratesticular testosterone concentrations [89,90]. Similarly, the oral selective estrogen receptor modulator clomiphene citrate is used off-label in men with low testosterone who wish to maintain fertility. It acts by blocking estrogenic feedback at the pituitary gland, increasing LH and follicle-stimulating hormone secretion, and thereby enhancing Leydig cell activity and testosterone synthesis while preserving or potentially improving sperm parameters [91].

Collectively, these therapeutic approaches highlight a central clinical principle: in men of reproductive age, strategies that support or activate the existing Leydig cell population are preferable to replacing testosterone exogenously, since the latter approach suppresses gonadotropin secretion and jeopardizes fertility [92].

## 5. Heterogeneity of Leydig Cells and Differential Stress Responses

### 5.1. Conceptual Framework and Translational Relevance

While Leydig cells have traditionally been regarded as a relatively uniform population of testosterone-producing cells, emerging single-cell and lineage-tracing studies indicate that this population is more heterogeneous than previously appreciated [93]. There appear to be distinct subpopulations of Leydig cells that differ in developmental origin, stage of differentiation, and functional capacity, including fetal versus adult Leydig cells and precursor versus fully mature steroidogenic cells [94]. This heterogeneity suggests that specific subsets of Leydig cells may respond differently to stressors such as toxicants or aging. Notably, this question has been studied primarily in animal models and remains underexplored in humans [95]. A deeper understanding of Leydig cell subtypes is likely to be crucial for the design of targeted therapies and regenerative strategies, since an intervention that protects or restores one subpopulation may have limited effects on others [96]. However, because much of the mechanistic insight into Leydig cell heterogeneity stems from rodent models, it remains to be determined how fully this complexity translates to humans. Translational research must clarify which rodent findings hold true in the human testis to guide realistic regeneration strategies.

### 5.2. Developmental Origins and the Stem/Progenitor Leydig Cell Pool

One important dimension of Leydig cell heterogeneity arises from developmental biology. In mammals, a population of fetal Leydig cells operates during fetal and early neonatal life, while a distinct lineage of adult Leydig cells differentiates at puberty and persists throughout adulthood [38,97,98]. In the adult testis, it has long been proposed that a reserve of progenitor or stem Leydig cells is present. These stem Leydig cells consist of undifferentiated mesenchymal-like cells located within the interstitium, capable of proliferating and giving rise to new Leydig cells, particularly in response to depletion or injury [99,100]. Evidence from rodent models has demonstrated that when adult Leydig cells are ablated by toxic injury such as ethane dimethanesulfonate exposure, the testis is able to regenerate a complete new population of Leydig cells [101]. This regenerative response depends on activation and differentiation of the resident stem or progenitor pool. Such findings imply that at least two subpopulations coexist in the adult testis: a differentiated cohort that sustains ongoing testosterone production and a quiescent precursor pool that can be mobilized when needed [102]. Rodent testes thus demonstrate a remarkable capacity to regenerate Leydig cells via this stem cell pool. By contrast, the adult human testis has not been shown to regenerate lost Leydig cells to the same extent [95]. This suggests that the stem Leydig pool may be far less robust in men, an important translational consideration for regeneration strategies.

### 5.3. Single-Cell Transcriptomics and Molecular Definition of Leydig Cell Subsets

Recent advances in single-cell RNA sequencing have provided more direct support for Leydig cell heterogeneity. A single-cell analysis of adult mouse testes identified two distinct Leydig cell clusters under basal conditions. One cluster expressed elevated levels of mature steroidogenic markers such as Star, Hsd3b family genes, Hsd17b3, and INSL3. The second cluster displayed gene signatures characteristic of less differentiated or more proliferative states, including elevated insulin-like growth factor 1 (IGF1) and related growth factors. The investigators described these as mature Leydig cells and immature Leydig cells, reflecting a continuum of differentiation within the adult Leydig compartment [103]. Manipulating specific transcriptional regulators in that model shifted the balance between these subpopulations. Loss of Sox30 increased the proportion of mature Leydig cells, indicating that these subsets differ functionally and that gene regulatory programs can influence their relative abundance [103]. Although this work was conducted in mice, the underlying concept likely applies to humans. Human testicular single-cell transcriptomic atlases have revealed age-dependent variation in Leydig cell gene expression and suggested the presence of multiple Leydig cell subsets across developmental and adult stages [96]. A recent analysis of human testes across the lifespan reported distinct shifts in Leydig cell transcriptional profiles between younger and older individuals, consistent with dynamic changes in subpopulation composition [72]. Whether the human adult testis maintains a stem Leydig cell pool as robustly as rodents remains unresolved. However, the identification of putative stem cell markers in a small subset of interstitial cells indicates that a regenerative or progenitor-like component may indeed exist in humans [99]. From a clinical standpoint, single-cell atlas data imply that certain Leydig subpopulations may be especially vulnerable or resilient to aging and toxicants, depending on their stress response profiles. In addition, shifts in the expression of niche signaling factors such as IGF1 or inflammatory cytokines among these subsets could explain how the testicular microenvironment changes with age or disease. The distinct marker genes defining each subset also offer potential biomarkers of Leydig cell health, which may enable earlier detection of testicular dysfunction and guide targeted interventions.

### 5.4. Differential Stress Responses and Niche Dependence Across Leydig Subpopulations

Leydig cell heterogeneity has important implications for infertility research because distinct subpopulations may differ in their vulnerability or resilience to physiological and environmental stressors. Certain progenitor-like Leydig cells may respond to environmental chemicals in ways that are fundamentally different from the responses of fully differentiated cells. An immature or stem-like Leydig cell may activate or proliferate following injury, whereas a mature Leydig cell may undergo apoptosis under the same conditions [1,104]. In the context of aging, it has been proposed that the pool of stem or less-differentiated Leydig cells becomes depleted or enters a dysfunctional state of quiescence, resulting in inadequate replacement of aged Leydig cells. According to one hypothesis, the age-related decline in testosterone production is partly driven by an exhaustion of the regenerative potential within the Leydig cell lineage. In this model, the progenitor cells may still be present, yet the aging testicular niche fails to support their differentiation, or the progenitors themselves gradually accumulate cellular damage as observed in aging rodent models [105].

Experimental evidence supports this concept. In aged mice, for instance, increased testicular tissue stiffness disrupts the stem Leydig cell niche, triggering oxidative damage and mitochondrial dysfunction in these progenitor cells and ultimately lowering testosterone output [106]. These findings underscore the importance of the extracellular environment and biomechanical cues in regulating Leydig cell renewal. They also suggest that targeting testicular fibrosis or oxidative stress might help preserve Leydig cell function, although such interventions remain untested in humans.

### 5.5. Functional Consequences for Endocrine Output and Paracrine Support of Spermatogenesis

Heterogeneity also has functional implications. If only a subset of Leydig cells is responsible for producing the majority of testosterone, loss of that subpopulation due to toxic exposure or disease could cause a substantial decline in androgen levels even when other Leydig cells remain intact. There is growing interest in determining whether specific Leydig cell subsets contribute more directly to fertility by supporting spermatogenesis through localized interactions, as suggested by findings in both rodent models and human single-cell studies [93,101]. Some single-cell analyses have shown that Leydig cells express paracrine mediators such as IGF1, cytokines, and growth-modulating factors that influence adjacent Sertoli or germ cells. If certain Leydig cell subtypes specialize in delivering these supportive signals, their dysfunction may have targeted and disproportionate consequences for the spermatogenic process [107,108]. In practical terms, if a toxicant or disease selectively impairs the subset of Leydig cells that provides key paracrine support, the result could be local germ cell dysfunction or infertility even if overall testosterone levels remain near normal.

## 6. Crosstalk Between Leydig Cells and Other Testicular Cells

The testis is not a simple collection of isolated cell types. It is a highly coordinated organ in which multiple cell populations communicate and support one another’s functions. Leydig cells, in particular, engage in intensive crosstalk with Sertoli cells and germ cells, forming an integrated system that sustains spermatogenesis [109]. Disruptions in this communication network can therefore produce cascading effects on fertility [110]. A classic example of this interdependence is that Sertoli cells require testosterone produced by Leydig cells in order to maintain sperm production, while Leydig cells depend on signals from Sertoli cells for their development and for optimal function [111,112]. In what follows, key aspects of these interactions are outlined, together with ways in which Leydig cell dysfunction may disturb the overall testicular environment. This framing is also clinically relevant, since endocrine and spermatogenic phenotypes often reflect combined failure across compartments rather than a single cellular defect.

### 6.1. Direct Leydig and Sertoli Cell Crosstalk

Sertoli cells, often described as the nurse cells of the seminiferous tubules, and Leydig cells, the principal interstitial endocrine cells, are linked by a bidirectional relationship [113]. Leydig cells secrete testosterone and INSL3, which then reach Sertoli cells through diffusion and circulation [4]. Testosterone binds to androgen receptors in Sertoli cells and enables them to support meiosis and spermiogenesis, as demonstrated by in vivo genetic models where disruption of Sertoli cell androgen signaling leads to severe spermatogenic failure [114]. When intratesticular testosterone levels are insufficient, spermatogenic output declines sharply, a pattern evident in both clinical hypogonadism and experimental suppression of Leydig cell activity [115]. This pattern is evident in clinical hypogonadism and in experimental models where Leydig cell activity is suppressed, leading to arrest of spermatogenesis at early stages because androgen stimulation is lacking [115].

In the opposite direction, Sertoli cells produce mediators that influence Leydig cell biology. Desert hedgehog (DHH) is a well-characterized Sertoli-derived morphogen that is essential for fetal and adult Leydig cell development [112,116]. During fetal and pubertal stages, DHH produced by Sertoli cells drives precursor cells toward the adult Leydig cell fate by specifying interstitial progenitors and supporting their steroidogenic differentiation [112,116]. In adulthood, Sertoli cells secrete a range of growth factors, estradiol generated through aromatization of testosterone, and various cytokines that collectively modulate Leydig cell steroidogenesis [109,117]. Among these, factors such as IGF1 and transferrin have been proposed to enhance Leydig cell function, whereas activin appears to exert more complex regulatory effects on Leydig cell steroidogenesis and somatic cell proliferation [118,119].

Recent work using three-dimensional testis-on-a-chip systems has highlighted the importance of spatial proximity and dynamic communication between Sertoli and Leydig cells [120]. When co-cultured in a microfluidic three-dimensional environment, these two cell types support each other’s survival and function far more effectively than when cultured separately, underscoring the requirement for intact paracrine loops in an in vitro platform [120]. In such models, toxicant exposure induces shared responses in both cell types and has led to the identification of biomarkers, such as serpin family B member 2 (SERPINB2), that are upregulated concurrently in Sertoli and Leydig cells [120]. These findings emphasize that the two cell types mount coordinated stress responses.

Disruption of this crosstalk can trigger a cascade of reproductive abnormalities. Damage to Leydig cells that reduces testosterone output or dysfunction of Sertoli cells that diminishes their supportive signals toward Leydig cells can both impair spermatogenesis [109,115]. Disturbed Sertoli–Leydig communication has been associated with developmental disorders including cryptorchidism and hypospadias, as well as adult conditions such as defective spermatogenesis and hypogonadism within the broader framework of testicular dysgenesis syndrome and Leydig cell-centric pathology [110,121]. Leydig cell dysfunction therefore rarely acts in isolation. Its ultimate impact on fertility is partly determined by how Sertoli cells, and thus germ cells, respond to altered signaling [109]. If Leydig cells produce suboptimal levels of testosterone or INSL3, Sertoli cells may fail to drive full germ cell differentiation even if other components of the system appear intact [121,122].

There is also growing evidence that distressed Leydig cells can send paracrine danger signals to Sertoli cells. Under conditions such as toxicant exposure or oxidative stress, Leydig cells may release cytokines and other mediators that adversely affect Sertoli cell health and blood–testis barrier integrity [123,124,125]. This could help explain why certain environmental insults simultaneously compromise both Leydig and Sertoli cells and consequently lead to a dual pattern of endocrine and spermatogenic failure [51,126]

### 6.2. Indirect Leydig and Germ Cell Crosstalk

Direct communication between Leydig cells and germ cells is less prominent than the dialog between Leydig and Sertoli cells, because germ cells reside within the seminiferous tubules behind the blood–testis barrier [111]. The main route of influence from Leydig cells to germ cells is hormonal. Adequate testosterone production by Leydig cells is essential for completion of meiosis and for final sperm maturation within the tubules, as supported by both experimental data and clinical observations linking low intratesticular testosterone to spermatogenic arrest [109,115].

In general, germ cells do not provide strong direct feedback to Leydig cells under physiological conditions. However, pathological situations can alter this balance. In syndromes characterized by complete germ cell loss, such as Sertoli cell-only syndrome, Leydig cells often become hypertrophic and more numerous, indicating altered trophic stimulation and local paracrine feedback [110]. This likely reflects altered hormonal feedback, with low inhibin secretion and elevated gonadotropins leading to chronic overstimulation of Leydig cells [110]. Thus, the status of germ cells can influence the endocrine environment indirectly through changes in Sertoli cell hormone production and pituitary feedback loops.

Some investigators have speculated that germ cells may release extracellular vesicles or exosomes that cross the tubular boundary and reach the interstitium, potentially carrying regulatory molecules to Leydig cells, although this remains hypothetical. A more established mode of indirect communication involves peritubular myoid cells and other somatic components. Peritubular cells surround the tubules and secrete factors such as platelet-derived growth factor (PDGF) and transforming growth factor beta (TGF-β) that act on both Sertoli and Leydig cells, thereby helping to coordinate tubular and interstitial function [109,127]. In this way, signals linked to germ cell status can be transmitted to the interstitium and influence Leydig cell behavior.

Taken together, direct germ cell–to–Leydig cell signaling remains incompletely defined, but the overall state of spermatogenesis feeds back on Leydig cells through a broader regulatory network that includes Sertoli cells, peritubular cells, and the hypothalamic–pituitary axis [109,115].

### 6.3. Implications for the Testicular Microenvironment and Therapy

Given the centrality of intercellular interactions within the testis, any comprehensive assessment of Leydig cell dysfunction in infertility should consider the entire testicular microenvironment [109,110]. Age-related decline in male fertility, for instance, is unlikely to be driven solely by Leydig cell aging or solely by Sertoli cell aging; rather, it more plausibly reflects parallel deterioration of both compartments and progressive disruption of their coordinated crosstalk [72,115,128]. Consistent with this concept, single-cell RNA sequencing analyses of testes from older men have reported aging-associated remodeling in both Sertoli and Leydig cells, including alterations in pathways governing cell–cell communication and, notably, reductions in outgoing signaling from Leydig cells and peritubular myoid cells [72,115]. Collectively, these observations support the view that fertility-oriented interventions in older males may need to target both tubular and interstitial compartments, rather than focusing on one cell type in isolation.

From a therapeutic perspective, this integrated view has two immediate implications. First, while supplemental testosterone can improve systemic androgen-deficiency symptoms, it is unlikely to restore spermatogenesis and may suppress gonadotropin secretion, thereby limiting fertility [129,130]. Moreover, transcriptomic signatures of Sertoli cell aging are consistent with reduced plasticity and altered support functions, suggesting that simply raising circulating testosterone may be insufficient to normalize the somatic environment required for germ cell maturation [115]. Second, attempts to “rejuvenate” Sertoli cell function are unlikely to be effective if Leydig cell endocrine support remains inadequate, including insufficient testosterone and impaired INSL3 signaling [115,121,122].

In practical terms, these insights are driving interest in combined or integrative interventions. One conceptual approach involves co-culture or co-transplantation strategies, in which mixtures of Leydig and Sertoli cells, or organoids containing both cell types, are introduced into testes that have lost endogenous function [109,120]. Another avenue focuses on improving the testicular niche. Enhancing testicular vascular health can improve oxygen and nutrient delivery to both Leydig and Sertoli cells and may reduce oxidative stress, while anti-inflammatory strategies might restore more favorable conditions for intercellular communication, since chronic inflammation is known to disrupt signaling between Sertoli and Leydig cells and to impair their supportive roles during aging [123,126,131].

Overall, Leydig cell dysfunction can initiate a feed-forward cascade in which diminished androgen output weakens Sertoli cell support and ultimately impairs germ cell development [109,110]. For this reason, a comprehensive fertility-preserving strategy should aim not only to correct a single cellular deficit, but also to re-establish the dialog between Leydig cells, Sertoli cells, and the germinal epithelium within an appropriately supportive microenvironment.

## 7. Emerging and Future Therapeutic Strategies

Therapeutic development for Leydig cell dysfunction is increasingly oriented toward two complementary objectives. The first is to restore physiological steroidogenic capacity and paracrine support within the interstitial compartment. The second is to preserve intratesticular androgen exposure in order to maintain spermatogenesis. This distinction has direct clinical relevance because exogenous testosterone may alleviate hypogonadal symptoms while suppressing gonadotropin secretion and lowering intratesticular testosterone, ultimately impairing sperm production. Evidence supporting this paradigm is provided by contemporary clinical studies and reviews [132,133,134]. In this context, current strategies increasingly emphasize Leydig cell-directed cytoprotection, fertility-preserving endocrine stimulation, interventions targeting senescence and metabolic vulnerability, and regenerative or gene-based approaches designed to reconstitute endogenous Leydig cell function rather than circumvent it.

### 7.1. Patient Stratification and Biomarker-Guided Therapy

Patient stratification is increasingly recognized as crucial when selecting among emerging therapies directed at Leydig cells. Key biomarkers help delineate the underlying defect and guide treatment choices. INSL3, secreted constitutively by mature Leydig cells, serves as an index of Leydig cell differentiation and functional reserve independent of acute LH fluctuations [3]. Baseline gonadotropin levels and sex hormone-binding globulin (SHBG) provide additional context by distinguishing primary testicular failure from secondary hypogonadotropic dysfunction and by reflecting metabolic influences on androgen bioavailability [135]. Clinical phenotype markers such as obesity and insulin resistance further inform therapy; for example, obesity-associated secondary hypogonadism is often characterized by low gonadotropin drive and low SHBG, a profile that may preferentially respond to weight loss or clomiphene rather than direct Leydig cell interventions [136].

As precision medicine advances, single-cell transcriptomic analyses are revealing distinct subpopulations of human Leydig cells [93]. In the future, such molecular signatures could help tailor interventions by identifying whether an individual’s Leydig dysfunction is driven more by differentiation failure, metabolic stress, or inflammatory senescence. This information could guide the choice of targeted therapy [72]. For instance, one patient’s profile might suggest using antioxidants or metabolic modulators, whereas another might benefit more from senolytic treatment, thereby matching the intervention to the underlying Leydig cell phenotype. Although this approach remains speculative, it underscores the potential of biomarker-guided selection in maximizing the efficacy of emerging treatments [96].

### 7.2. Enhancing Leydig Cell Resilience: Cytoprotection and Translational Platforms

Although exposure reduction remains the primary preventive measure, there is growing emphasis on approaches that increase Leydig cell resilience by limiting oxidative injury, stabilizing mitochondrial function, and correcting maladaptive stress responses. Preclinical studies indicate that selected bioactive compounds can partially preserve steroidogenesis in toxicant-driven injury models. Mangiferin attenuated DEHP-associated testicular injury in rats with preservation of steroidogenic function in vivo, supporting a cytoprotective mechanism that is consistent with antioxidant and antiapoptotic activity [57]. Icariin has also been reported to counter phthalate-associated suppression of steroidogenic pathways in experimental systems [137]. In vitro work suggests that antioxidant-rich extracts may protect Leydig steroidogenesis under toxic or metabolic stress, including *Sambucus nigra* extract in TM3 Leydig cells [138]. Rutin has similarly been associated with recovery of testosterone output after oxidative injury in Leydig cell models [54]. These data collectively support biologic plausibility, yet they remain dominated by rodent studies and immortalized cell lines. Translation requires careful attention to dosing, tissue distribution, endocrine endpoints, and potential off-target effects before clinical recommendations can be justified.

A complementary direction is the use of targeted metabolic substrates or micronutrients to stabilize steroidogenic transcriptional programs in human Leydig systems under stress. L-cysteine has been shown to upregulate testosterone biosynthesis pathways and related gene programs in cultured human Leydig cells [139]. Such results are best interpreted as proof of concept and require replication in primary human Leydig preparations and under clinically relevant exposure patterns, including mixed endocrine disruptor mixtures and metabolic lipotoxicity states, prior to inference about clinical efficacy.

Therapy discovery is also being accelerated by human-relevant platforms that preserve multicellular organization and intercompartmental signaling. Microfluidic testis-on-a-chip systems have demonstrated coordinated Sertoli–Leydig stress responses and have identified candidate biomarkers such as SERPINB2 under toxicant exposure [120]. Related microphysiological approaches for modeling human testicular function ex vivo further increase translational fidelity [140]. Multi-cellular organoid and chip approaches incorporating spermatogonial stem cells together with Sertoli and Leydig compartments are also emerging as enabling platforms for mechanistic screening and biomarker development [141].

### 7.3. Lifestyle and Fertility-Preserving Endocrine Therapies

Among currently deployable interventions, metabolic optimization is the most immediately actionable strategy to improve Leydig output at a population level, particularly in obesity-related functional hypogonadism. Contemporary synthesis suggests that weight loss tends to increase circulating testosterone through improvements in insulin sensitivity, inflammatory tone, and aromatase-mediated androgen conversion [78]. However, endocrine recovery does not uniformly translate into improved semen parameters. Future studies should integrate reproductive endpoints such as sperm concentration, total motile count, and sperm DNA fragmentation alongside endocrine measures to identify which clinical phenotypes experience fertility benefit.

For men with symptomatic low testosterone who wish to preserve fertility, endocrine stimulation is generally preferred over testosterone replacement because it supports endogenous intratesticular androgen production. Human chorionic gonadotropin is a key option as an LH analog that stimulates Leydig steroidogenesis [142]. Contemporary clinical series indicate that human chorionic gonadotropin (hCG) monotherapy can increase testosterone and improve symptoms in selected cohorts, including men previously exposed to exogenous testosterone [90,143]. In testosterone- or anabolic steroid-associated infertility, combined gonadotropin regimens are used to facilitate spermatogenic recovery, and recent reports support efficacy in carefully selected patients [144]. These data support treatment algorithms that distinguish men seeking symptom control from those requiring fertility preservation or restoration, because endocrine goals and trade-offs differ substantially.

Selective estrogen receptor modulators, particularly clomiphene citrate, remain widely used off-label to increase gonadotropin drive and thereby stimulate Leydig testosterone production while preserving spermatogenesis. A systematic review summarizes efficacy and safety across available studies [145]. A recent meta-analysis of randomized trials further evaluates endocrine effects of clomiphene and enclomiphene [146]. In parallel, systematic reviews and meta-analyses indicate that modulation of estrogen pathways can influence semen parameters in men with secondary hypogonadism, although heterogeneity is substantial and patient selection remains critical [147]. Progress in this domain will depend on identifying predictors of response such as baseline gonadotropins, SHBG, estradiol, testicular volume, and markers of Leydig reserve. The goal is to enable endocrine stimulation guided by mechanistic insight rather than empirical use.

### 7.4. Senescence-Directed and Metabolic Rejuvenation Strategies

Leydig decline with aging is increasingly conceptualized as a convergence of mitochondrial stress, inflammaging, and cellular senescence. This framing motivates senescence-directed interventions as candidates for late-onset hypogonadism and age-associated infertility risk [68,148]. Rodent proof-of-concept studies indicate that reducing senescent cell burden can improve aspects of testicular endocrine function. FOXO4-DRI, designed to disrupt FOXO4–p53 interaction in senescent cells, improved the testicular microenvironment and alleviated age-related testosterone insufficiency in an aging mouse model [149]. More broadly acting senolytic combinations, such as dasatinib plus quercetin, increased serum testosterone and improved sperm concentration in aged male mice, although fertility outcomes did not necessarily normalize, emphasizing the need for endpoints that reflect both endocrine recovery and reproductive competence [150]. Clinical translation remains speculative, requiring resolution of safety, tissue specificity, and the physiological roles of senescent cells in the testis.

A complementary strategy is restoration of Leydig metabolic competence. Impaired ketogenesis has been identified as a driver of testicular aging, and increasing ketone body availability, including oral beta-hydroxybutyrate supplementation, reduced Leydig senescence and improved testosterone output in aged mice [67]. These findings link endocrine aging to substrate utilization and epigenetic metabolic signaling, and they motivate careful evaluation of whether testicular ketone signaling can be modulated in humans without unacceptable systemic effects. Any senolytic intervention will also require testis-specific delivery to avoid harm in other tissues. Systemically administered senolytic drugs such as dasatinib carry risks of unintended cell depletion and organ toxicities, and no clinical studies have yet evaluated their use for male reproductive aging. These approaches remain confined to animal models, highlighting the significant preclinical gap before human translation can be considered [150,151].

### 7.5. Regeneration, Transplantation, and Gene-Based Repair of Leydig Function

Regenerative strategies aim to restore a functional Leydig population capable of regulated testosterone production within an appropriate niche. A decellularized testicular extracellular matrix hydrogel has been shown to support stem Leydig expansion and steroidogenic differentiation, addressing the concept that an aged microenvironment can be hostile to Leydig renewal [152]. In parallel, stem Leydig cells have been reported to support macrophage immune homeostasis through mitochondrial transfer in a TRPM7-dependent manner in a torsion and acute injury model, indicating that successful regeneration may require coordinated restoration of immune niche function [153].

Cell replacement has advanced through improved differentiation protocols. Cells resembling Leydig cells, derived from human-induced pluripotent stem cells (iPSCs), demonstrate robust steroidogenic gene expression and sustained testosterone secretion [154]. Earlier directed differentiation approaches using NR5A1-driven programming also provide proof of concept [155]. Encapsulation offers a partial solution to immune rejection. In one study, human Leydig-like cells encapsulated in alginate–polylysine–alginate microcapsules showed prolonged survival and partial testosterone restoration in castrated mice without immunosuppression [156]. At present, many of these approaches address androgen replacement more directly than fertility restoration, and achieving meaningful spermatogenic benefit will likely require functional integration within the native testicular microenvironment. These regenerative cell therapies remain far from clinical application. Ensuring their long-term safety is paramount, since transplanted cells could form tumors or provoke immune reactions if not rigorously controlled [157]. Moreover, delivering cells into the human testis and achieving durable engraftment without disturbing native tissue architecture are major challenges that must be overcome before any clinical translation [158].

Gene therapy is particularly compelling for monogenic Leydig failure. Interstitial adeno-associated virus (AAV)-mediated Lhcgr delivery restored testosterone production and enabled fertility in Lhcgr-deficient mice [159]. Subsequent work reported restoration of natural fertility and improvement of systemic phenotypes, supporting durability [160]. Hybrid approaches that combine ex vivo gene correction of stem Leydig cells with transplantation have also achieved restoration of Leydig function and fertility in disease models [161]. Although translation will require stringent safeguards to minimize off-target transduction and prevent germline exposure, these studies establish feasibility and define mechanistic benchmarks for future clinical development. However, human translation of such gene therapies remains distant and will require stringent safeguards to avoid transduction of unintended cells or germline exposure. Achieving efficient delivery of genes to Leydig cells without provoking immune responses is a major challenge. Even vectors like AAV, which have relatively low immunogenicity, can trigger neutralizing antibodies or cause rare insertional mutations [162]. Rigorous preclinical safety studies in large animal models will be required to address these risks before clinical trials can be considered. Pathway-targeted small molecules remain attractive for scalability, and recent syntheses highlight cholesterol transport and mitochondrial steroidogenic machinery as actionable nodes for drug discovery [163]. Key mechanistic hubs, representative readouts/biomarkers, and mechanism-informed opportunities to preserve or restore Leydig cell function are summarized in Table 1.

## 8. Conclusions

Leydig cells are central to the endocrine axis of male reproduction, yet their dysfunction remains an under-recognized contributor to male infertility. Converging evidence indicates that contemporary exposures and conditions, including endocrine disrupting chemicals, obesity with metabolic dysregulation, and population aging, are associated with impaired Leydig cell steroidogenesis and reduced paracrine support. In parallel, advances in single-cell and systems-level approaches show that Leydig cells are heterogeneous and embedded within a tightly regulated intercellular network with Sertoli, peritubular, immune, and germ cell compartments. Together, these observations support the view that Leydig cell health helps shape the intratesticular milieu required for efficient spermatogenesis. Clinically, this framework supports a more integrated infertility work-up that includes evaluation of Leydig cell function alongside standard endocrine semen assessment. It also supports fertility-preserving strategies that optimize endogenous androgen action.

Future priorities include defining mechanistic nodes that link cellular stress responses to steroidogenic failure. Priority pathways include autophagy and mitochondrial quality control, senescence programs, and metabolic regulators. These insights should be translated into actionable biomarkers and targeted interventions. Carefully designed translational studies and clinical trials are needed to determine whether antioxidant, metabolic, endocrine stimulatory, regenerative, or gene-based approaches can improve hormonal endpoints and reproductive outcomes without compromising spermatogenesis. The evidence is strongest for Leydig cell steroidogenic vulnerability in the context of aging and metabolic dysfunction, and for chemical toxicants that perturb steroidogenic signaling in experimental systems. A unifying hypothesis is that toxicant exposures accelerate stress and metabolic pathways that are already engaged by aging and metabolic dysfunction, thereby lowering the threshold for Leydig cell failure in susceptible men. Testing this integrative model will require longitudinal human studies that link exposures to deep phenotyping and trials that evaluate mechanism-aligned interventions. Until such data are available, Leydig cell dysfunction is best viewed as a biologically grounded and potentially modifiable target within precision andrology. Its relevance for hormonal health is clear, whereas the extent to which targeting Leydig cell pathways improves fertility remains to be established.

## Figures and Tables

**Table 1:** Mechanism-informed opportunities to preserve or recover Leydig cell function in aging and acquired stress states.

Mechanistic Hub/Process	Representative Readouts	Mechanism-Informed Opportunities	Evidence Level
Mitochondrial dysfunction and redox stress	Clinically measurable. Testosterone, LH, the LH testosterone relationship, INSL3 [3]. Mechanistic. ROS and lipid peroxidation, glutathione depletion, mitochondrial fission, reduced steroidogenic enzyme activity [42].	Exposure mitigation. Antioxidant and mitochondrial support, for example, rutin [54], icariin [55,56], mangiferin [57]. Metabolic optimization and weight loss [78,79,83].	Mixed
ER stress/unfolded protein response	UPR activation. GRP78/BiP, CHOP, impaired folding of steroidogenic enzymes [33]. Clinically measurable. Testosterone, LH, the LH testosterone relationship, INSL3, steroid precursor profiles [12].	Reduce ER stress inducing exposures, for example, tributyltin chloride [33]. Optimize metabolic and inflammatory milieu [68]. Explore ER stress modulators in preclinical settings [33].	Animal and cell line
Impaired autophagy/mitophagy	Beclin 1, LC3 II, p62. PINK1, Parkin. Accumulation of damaged mitochondria and reduced steroidogenic capacity [34].	Support organelle quality control, for example, vitamin D3 and VDR-linked autophagy [35], resveratrol and ATG7-related pathways [30]. Mitigate mitophagy inhibition in toxicant exposure, for example, cadmium [34].	Mixed
DNA damage response/genomic stress	DNA damage markers. γH2AX, oxidative DNA lesions, p53, p21. Clinically measurable. γH2AX in testicular tissue when available [27,40].	Lower redox and toxicant burden. Target upstream oxidative stress and inflammation. Develop biomarkers to risk stratify vulnerability [123].	Mixed
Apoptosis and cell loss	Bax, caspase 3, TUNEL. Reduced Leydig cell number and reserve [27]. Clinically measurable. Low INSL3, high LH relative to testosterone, steroid precursor patterns [12].	Cytoprotection by limiting mitochondrial injury and oxidative stress. Consider fertility-preserving endocrine stimulation rather than exogenous TRT when indicated [132,133,134].	Human and animal
Senescence programs	p16, p21, senescence-associated beta galactosidase. SASP-related inflammation and reduced steroidogenic capacity [149]. Clinically measurable. Low INSL3 with a rising LH testosterone relationship [3].	Senescence-directed strategies in preclinical models, for example, FOXO4-DRI [149], dasatinib plus quercetin [150]. Clinical readiness remains limited due to delivery challenges and the need for testis selective exposure. Off-target risks and long-term endocrine safety require careful preclinical evaluation.	Animal and cell line
Metabolic dysfunction/impaired ketogenesis	Obesity-related endocrinopathy with reduced Hmgcs2 and beta hydroxybutyrate signaling and FOXO3a activity [67]. Clinically measurable. Metabolic phenotype, beta hydroxybutyrate, testosterone, LH, INSL3 [14].	Weight loss and metabolic optimization [78,79,83]. Metabolic substrate strategies, for example, beta hydroxybutyrate supplementation in mice [67]. Clinical readiness remains limited, and systemic metabolic effects and timing of exposure are key translational variables.	Mixed
Niche remodeling and interstitial crosstalk	Interstitial inflammation and altered immune and vascular signaling. Increased ECM stiffness disrupting stem Leydig pool homeostasis [101].	Niche-targeted approaches, for example, ECM-based hydrogels supporting stem Leydig cells [152]. Improve vascular and inflammatory milieu [123,131]. Use testis on a chip platforms for translational screening [120].	Mixed
Regeneration and gene-based repair	Leydig failure with limited endogenous recovery and need for durable, regulated androgen production [159]. Clinically measurable. Testosterone, LH, the LH testosterone relationship, INSL3 [156].	Stem Leydig niche reconstruction [152]. iPSC-derived Leydig-like cells with stable testosterone secretion [154]. Encapsulated Leydig-like cell transplantation [156]. AAV-mediated Lhcgr therapy [159,160] and gene correction approaches [161]. Clinical readiness remains limited due to delivery, immunogenicity, dose control, and off-target risks, with cell-based strategies also requiring stringent tumorigenicity assessment.	Animal and cell line

Abbreviations: ER, endoplasmic reticulum; UPR, unfolded protein response; GRP78/BiP, glucose-regulated protein 78 (binding immunoglobulin protein); CHOP, C/EBP homologous protein; ROS, reactive oxygen species; SASP, senescence-associated secretory phenotype; ECM, extracellular matrix; ATG7, autophagy-related 7; LC3, microtubule-associated protein 1A/1B-light chain 3; PINK1, PTEN-induced kinase 1; TRT, testosterone replacement therapy; AAV, adeno-associated virus; iPSC, induced pluripotent stem cell; VDR, vitamin D receptor; LH, luteinizing hormone. INSL3, insulin-like peptide 3.

## Data Availability

No new data were created or analyzed in this study.
